# Brain Activity toward Gaming-Related Cues in Internet Gaming Disorder during an Addiction Stroop Task

**DOI:** 10.3389/fpsyg.2016.00714

**Published:** 2016-05-19

**Authors:** Yifen Zhang, Xiao Lin, Hongli Zhou, Jiaojing Xu, Xiaoxia Du, Guangheng Dong

**Affiliations:** ^1^Department of Psychology, Zhejiang Normal UniversityJinhua, China; ^2^Shanghai Key Laboratory of Magnetic Resonance, Department of Physics, East China Normal UniversityShanghai, China

**Keywords:** fMRI, internet gaming disorder, stroop, attentional bias

## Abstract

**Background and Aims:** Attentional bias for drug-related stimuli is a key characteristic for drug addiction. Characterizing the relationship between attentional bias and brain reactivity to Internet gaming-related stimuli may help in identifying the neural substrates that critical to Internet gaming disorder (IGD).

**Methods:** 19 IGD and 21 healthy control (HC) subjects were scanned with functional magnetic resonance imaging while they were performing an addiction Stroop task.

**Results:** Compared with HC group, IGD subjects showed higher activations when facing Internet gaming-related stimuli in regions including the inferior parietal lobule, the middle occipital gyrus and the dorsolateral prefrontal cortex. These brain areas were thought to be involved in selective attention, visual processing, working memory and cognitive control.

**Discussion and Conclusions:** The results demonstrated that compared with HC group, IGD subjects show impairment in both visual and cognitive control ability while dealing with gaming-related words. This finding might be helpful in understanding the underlying neural basis of IGD.

## Introduction

Internet gaming disorder (IGD) is rapidly becoming a prevalent mental health concern around the world over the last few decades (Dong et al., 2011). People often define IGD as an excessive or uncontrolled use of the internet-game followed by negative consequences (Beard and Wolf, 2001; Grant et al., 2010). IGD is also conceptualized as a “behavioral addiction” as its proposed diagnostic criteria are so closely parallel to substance use disorders and pathological gambling (Thaler and Shefrin, 1981; Targhetta et al., 2013; Thorens et al., 2014). Apart from considering substance-use and addictive disorders, the fifth edition of the Diagnostic and Statistical Manual of Mental Disorder (DSM-5) has generated criteria for IGD in the Section Results containing disorders warranting additional study in 2013 (Petry and O'Brien, 2013; Dong et al., 2015).

Researches have demonstrated that attentional bias is the key characteristic for addiction, which contributes to people's motivation to take addictive substance. Various theories of addiction have argued that addictive behaviors are characterized by attentional bias for substance-related stimuli (Noël et al., 2005; Fields, 2008). Moreover, measuring attentional bias toward substance-related cues may be a valid method to identify people with heightened relapse vulnerability (Schouw et al., 2013). A wealth of researchers from the past two decades have found attentional bias for substance-related stimuli (presented verbally, pictorially, or as *in vivo* exposure) in users of alcohol and a variety of other substances, including nicotine, cannabis, opiates, and cocaine (Thaler and Shefrin, 1981; Koob and Volkow, 2010). For example, smokers show longer RT in naming smoking-related words than non-smokers. Therefore, the smokers exhibited more attentional bias for smoking-related words compared with the non-smokers (Fields, 2008). The similar features have also been observed in pathological gamblers (Kertzman et al., 2006), a type of behavioral addiction. As behavioral addiction, similar results have been found that the reaction time in IGD subjects is longer than that of HC group, which means that an attentional bias in IGD subject and the attentional bias in IGD subjects can be used to identify addiction (Metcalf and Pammer, 2011).

Although no chemical or substance intake is involved in IGD, excessive use of internet games can also lead to physical dependence, eventually causing psychological, social, or work difficulties, similar to other addictions (Griffiths, 2000; Holden, 2001; Murali and George, 2007). The IGD is currently positioned in the appendix of the DSM-V as a condition requiring further study and positioned as a behavioral addiction (Kaptsis et al., 2016; Király et al., 2015). As a behavioral addiction, IGD may share similar neuropsychological (i.e., development of euphoria, craving, and tolerance) characteristics with other behavioral addictions (Dong et al., 2010, 2011, 2013a,b). Considering the similarities in symptoms, the present study is set to explore whether behavioral aspects in IGD are commonly related to those found in substance-dependent persons and pathological gambler.

We used the addiction Stroop task in our study. It is a modified version of the classical Stroop task and a most widely used test of substance-related attentional bias (Cox et al., 2006). The goal of this study was to explore the relation between the attentional bias toward gaming-related stimuli and brain activation to gaming-related cues. In this task, attentional bias is inferred if participants' performance on a primary task (e.g., color-naming) is impaired when substance-related stimuli (e.g., gaming-related words) are presented simultaneously. It is indexed as the difference between mean color-naming response time (RT) on trials with substance-related and RT with neutral ones. It is supposed that longer RT to the substance-related words indicates the automatic processing of the semantic contents of the words and impair the color naming, or results from the attempts to avoid elaborative processing of substance-related words (Thaler and Shefrin, 1981; Cox et al., 2006).

Except behavioral evidences, researchers have also investigated the neurobiological underpinnings of disorders. Functional magnetic resonance image (fMRI) studies have identified that a number of brain regions are associated with addictive substance-related stimuli (Schouw et al., 2013; Yuan et al., 2014; Yang et al., 2015). For example, previous studies have found altered default network in brain regions, namely inferior parietal lobule, middle frontal gyrus, middle temporal, cingulate gyrus (Lin et al., 2012; Ding et al., 2013; Kim et al., 2015). Beside, studies using voxel-based morphometry (VBM) technique have demonstrated multiple structural changes of brain in internet gaming disorder (IAD) and IGD subjects, such as the bilateral dorsolateral prefrontal cortex (DLPFC), the orbitofrontal cortex (OFC), middle temporal gyrus, insula and anterior cingulate (Yuan et al., 2011; Lin et al., 2015). Although recent studies have found the relation between the attentional bias and increased reactivity in the prefrontal cortex (Luijten et al., 2011; Ko et al., 2013), insula, the anterior cingulate cortex (Janes et al., 2010), the parietal gyrus and the temporal gyrus (Luijten et al., 2011), it still remains unknown whether these brain activation patterns are affected by attentional bias toward gaming-related words. Accordingly, in this study, we calculated the correlation between behavioral performance during the addiction Stroop task and the brain activation to gaming-related words vs. neutral words to find the potential relations between them.

IGD subjects with experience of playing online games would show automatic reaction toward gaming-related stimuli (Fields, 2008; Ko et al., 2015). Thus, they need to engage more cognitive resource in focusing their attention on the color rather than semantics of the gaming-related words to complete the task (coloring naming task). Therefore, we hypothesized that the IGD subjects would display different brain activities in regions responsible for enhanced efficiency of cognitive control, such as greater activation in the DLPFC (Ko et al., 2013).

## Methods

### Participants

Participants were university students and were recruited through advertisements. Participants were right-handed males and had no difficulty in naming color (19 IGD subjects, 21 healthy controls (HCs)). The IGD subjects and HC group did not significantly differ in age [IGD: 22.2 ± 3.1 years; HC: 22.8 ± 2.4 years; *t*_(38)_ = 0.694, *p* > 0.05]. Only males were included due to higher IGD prevalence in men than that of women (Siomos et al., 2008; Gentile et al., 2011; Kamal and Mosallem, 2013; Adiele and Olatokun, 2014; Baggio et al., 2015). Defense of the Ancients (Dota) is mod built on the Real-Time-Strategy (RTS) games Warcraft III and is one of the most popular online games (Loh and Soon, 2006). And, the IGD subjects all reported to play the Internet game named “Dota.” The IGD and HC participants did not meet DSM-V criteria for abuse of or dependence on substances, including alcohol and nicotine. All participants were medication free and were instructed not to use any substances of abuse, including coffee, on the day of scanning. Besides, all participants underwent structured psychiatric interviews (Lecrubier et al., 1997) performed by an experienced psychiatrist and all participants were free of active substance abuse, neuropsychiatric disorder and Axis I psychiatric disorder.

IGD participants met both Chinese IAT requirements: (1) spend more than 6 h online everyday aside from work, and (2) show symptoms such as psychological dependence, abstinent reaction, compulsive use, social withdrawal, or negative effect on body and mental health for more than 3 months. All IGD participants spent most of their time online playing internet games. HCs all scored lower than 30 on Young's IAT (mean = 28.5, *SD* = 12.0) and did not satisfy either of the Chinese IAT requirements, while the IAT scoring all over 60 ((mean = 64.4, *SD* = 10.3). And the scores in IGD subjects were significant higher than HC group (*t* = -8.944, *p*=0.000).

### Measures

The selection of IGD was based on Young's online internet addiction test (IAT) (Polezzi et al., 2008; Grant et al., 2010) scores of 50 or higher and at the same time, reach the criteria of the proposed 9-items IGD diagnostic scale based on DSM-V (Petry and O'Brien, 2013; Dong et al., 2015; Greenhow et al., 2015). Young's IAT consists of 20 items associated with online internet use including psychological dependence, compulsive use, withdrawal, related problems in school or work, sleep, family or time management. The IAT was proved to be a valid and reliable instrument that can be used to classify IAD (Widyanto and McMurran, 2004; Widyanto et al., 2011). For each item, a 5-point rating scale would appear on the screen and participants needed to choose from 1 to 5 (1 rarely, 5 always). Scores over 50 indicate occasional or frequent internet-related problems and scores over 80 mean “Your internet usage is causing significant problems in your life”) (www.netaddiction.com). To classify IGD, individuals with IAD also needed to respond positively to the following question: “you spend most of your online time playing online games (>80%) (Yes).”

### Procedure

**Figure 1:**
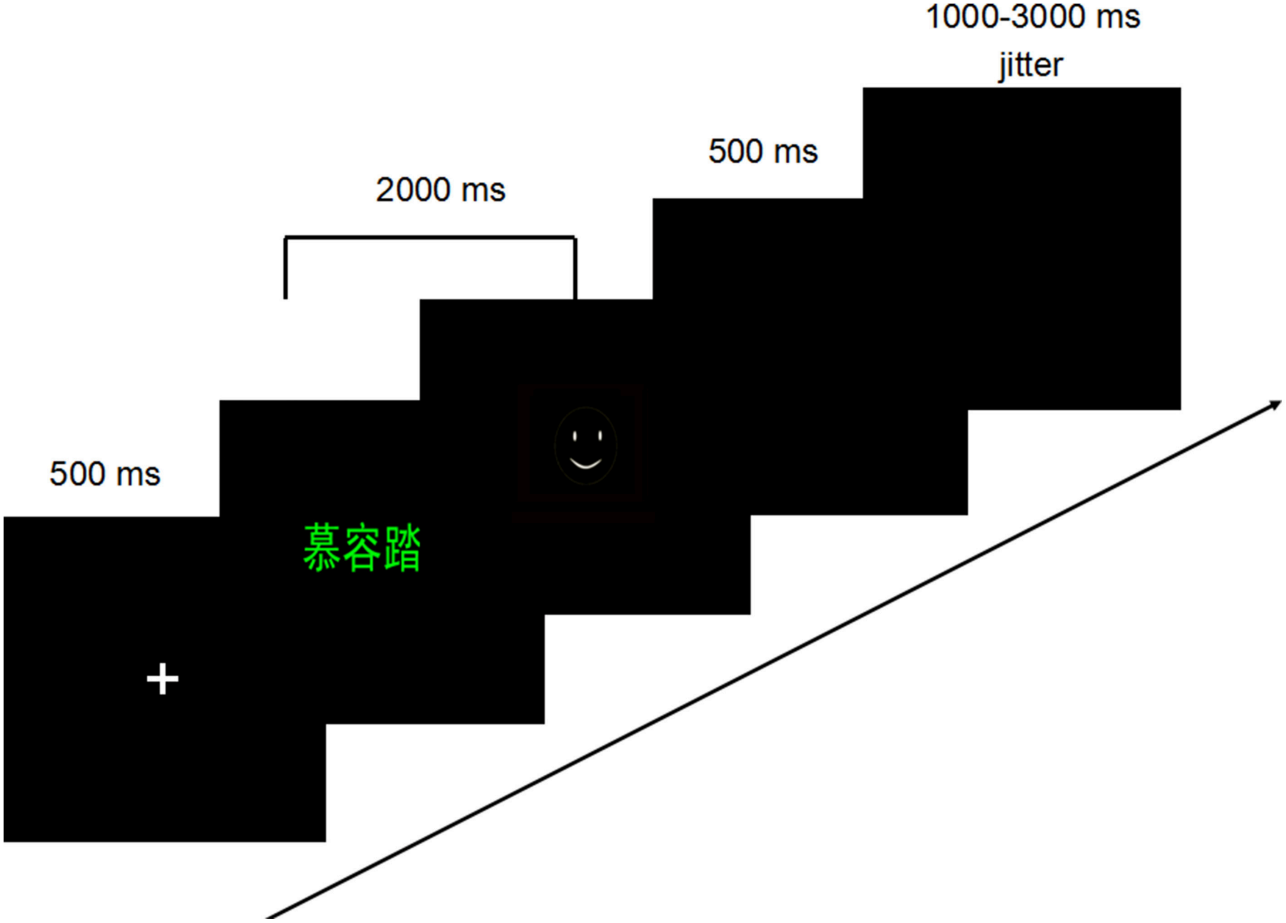
**The procedure of addiction Stroop task in present study**.

An addiction Stroop task was administered. Two kinds of words (see in the supplementary document), including 30 internet gaming-related and 30 matched control words (matched in terms of semantic properties that affect reading speed, including word length, number of syllables per word), were presented randomly in red, green and yellow. Moreover, to control the familiarity of the words between the two groups, before the study, the familiarity of words have been evaluated among people who have played the game “dota” and who never have played (they all did not participant in this study). So the familiarity of gaming-related words and neutral ones which used in this study has no difference. In present study, the gaming-related words were common names of different categories, such as weapons, characters and tips in Dota. And the neutral words were selected from names of common tools, supplies etc., which are matched with the categories of gaming-related words. On each trial, a single word will appear in one font color (red, yellow, or green), and the participant will be instructed to name the color as quickly and accurately as possible using three buttons (i.e., green = thumb, red = index finger, yellow = middle finger) of a three-button response box (invivocorp.com/). The task was composed of 120 trials where the gaming-related words and neutral words with certain font color appeared randomly (Supplementary Material 1). Each word appeared twice and the same words or the words with same color did appear continuously. In each trial (see Figure 1), the stimuli were presented which would disappear once the participants pressed the button, after presenting a cross in the center of the screen as fixation point for 500 ms. Besides, the RT of the stimuli plus the duration of a followed black screen are totally 2000 ms. Then a feedback would appear for 500 ms, the right response coming up with a smile face and a wrong one with a cry face. A black screen was presented for a random interval of 1000–3000 ms (average 2000 ms) between trials. Stimuli were presented and behavioral data were collected using E-prime software (Psychology Software Tool, Pittsburgh, PA.). A guaranteed 20 Yuan (about 3 dollars) would be given to participants.

### Statistical analysis

#### Image acquisition and pre-processing

Structural images covering the whole brain were acquired with a T1-weighted three-dimensional spoiled gradient-recalled sequence (176 slices, *TR* = 1700 ms, *TE* = 3.93 ms, slice thickness = 1.0 mm, skip = 0 mm, flip angle = 15°, inversion time 1100 ms, field of view = 240 × 240 mm^2^, in-plane resolution = 256 × 256). Functional MRI was performed on a 3 T scanner (Siemens Trio) with a gradient-echo EPI T2 sensitive pulse sequence in 33 slices (interleaved sequence, 3 mm thickness, *TR* = 2000 ms, flip angle 90°, field of view 220 × 220 mm^2^, matrix 64 × 64). Participants were instructed to view the stimuli, which were presented on a screen in the head coil, through Invivo synchronous system (Invivo Company, www.invivocorp.com/).

Imaging analysis was conducted using SPM8 (http://www.fil.ion.ucl.ac.uk/spm). Images were slice-timed, reoriented, and realigned to the first volume. T1-co-registered volumes were then normalized to an SPM T1 template resulting in an isometric voxel size of 3 × 3 × 3 mm^3^ voxels. Finally, images were smoothed with a 6 mm full-width at half maximum Gaussian kernel.

#### Data analysis

Response time and accuracy rate were computed with analysis of variance (ANOVAs) in two-by-two mixed ANOVAs separately, with group as a between-subject factor (IGD subjects; HC group) and word type as a within-subject variable (gaming-related words, neutral words). Besides, we also analyzed the attention bias (defined as the mean RT of the gaming-related words minus those of the neutral ones) between the two groups with an independent sample *t*-test.

For the fMRI data, pre-processed images were entered into a standard multiple regression (ordinary least squares) in NeuroElf (neuroelf.net), which included regresses for the conditions of interest. First, we examined the brain activation related to the main effect of group and words type separately. Then to assess the interaction effects, one-sample *t*-test was computed for the ((IGD Substance-related words - IGD Neutral words) - (HC Substance-related words - HC Neutral words)) contrast. For this contrast, we first identified clusters of contiguously significant voxels at an uncorrected threshold *p* < 0.05. Then we tested these clusters for cluster-level FWE (family wise error) correction *p* < 0.05 and the AlphaSim estimation indicated that clusters with 102 contiguous voxels would achieve an effective FWE threshold *p* < 0.05. Besides, we extracted the BOLD signal within each cluster that demonstrated between-group differences (for each ROI, a representative BOLD beta value was obtained by averaging the signal of all the voxels within the ROI) and entered these data for the IGD subjects into correlation analysis with attentional bias (defined as the mean RT of the gaming-related words minus those of the neutral ones).

### Ethics

All participants provided written informed consent. The experiment conformed to The Code of Ethics of the World Medical Association (Declaration of Helsinki). The Human Investigations Committee of Zhejiang Normal University approved this research.

## Results

### Behavioral performance

Error trials (in each participant: mean = 2.54, *SD* = 2.93) were excluded from the further analysis. To reduce the influence of outliers, 0.83% trials (mean = 1.00, *SD* = 1.92; the RT less than 300 ms or more than 1400 ms) were removed in order to eliminate anticipatory responses with no response or pressing by mistaken. And the number of trials excluded has no significant difference between the two groups (*F*=2.634, *p*=0.224).

Firstly, RT and accuracy rate were conducted with two factors repeated measures ANOVAs separately (see Table 1). The words type (gaming-related words vs. neural words) was a within-subject variable and group (IGD subjects vs. HC group) was a between-subject variable. For RT, this analysis revealed a main effect of the word [*F*_(1, 38)_=4.558, *p*=0.039] with the mean RT of gaming-related words being shorter, compared with neutral ones for all participants together. The interaction effect was not significant [*F*_(1, 38)_=0.432, *p* = 0.515]. Although IGD subjects showed longer RT than the HC group, it did not reach statistical significance [*F*_(1, 38)_=1.527, *p*=0.224]. For accuracy rate, no main effects or interaction effect [*F*_(1, 38)_=0.005, *p*=0.945] were found. The IGD and HC group did not significantly differ on accuracy rate [*F*_(1, 38)_=0.143, *p*=0.708]. And the accuracy rate for gaming-relate words and the neutral one showed no significant difference [*F*_(1, 38)_=2.718, *p*=0.107]. In addition, to compare the attention bias between these two groups, an independent sample *t*-test was performed. The results revealed that the IGD subjects showed more obvious attentional bias than the HC group, although it did not reach statistical significant [*F*_(1, 38)_=2.352, *p*=0.133].

**Table 1:** **Behavioral results of the Stroop task**.

		**RT (ms)**		**Accuracy rate**	
		**Neutral**	**Gaming-related**	**Neutral**	**Gaming-related**
HC	*M*	622.038	642.040	0.978	0.986
	*SD*	119.850	113.997	0.024	0.027
IGD	*M*	579.301	590.622	0.975	0.984
	*SD*	101.600	105.421	0.041	0.027

M, arithmetic mean; SD, standard deviation; HC, healthy control group; IGD, internet gaming disorder.

### Imaging data

First, we found the fMRI signals in the left and right postcentral gyrus (Brodmann's areas (BAs) 2/3) and left temporal gyrus (BA 37) were significantly decreased for IGD subjects compared with HC group. We next determined the main effect of word type in brain regions in inferior frontal gyrus (BA 45) and the right pre-central gyrus (BA 6), with significant increased activation in gaming-related words than neutral words.

**Figure 2:**
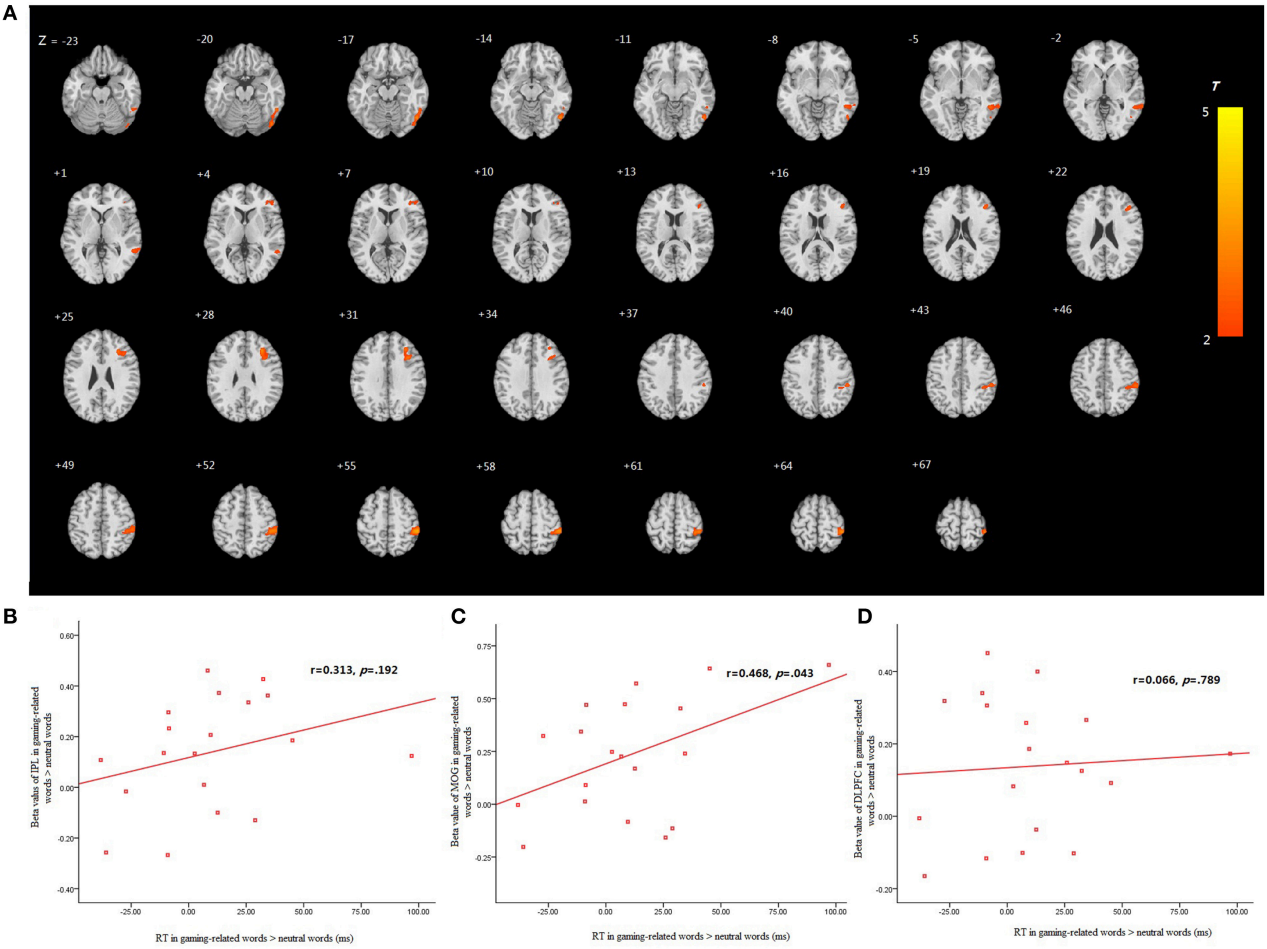
**Brain regions showing significant activation in the comparison of ((IGD Substance-related words - IGD Neutral words) - (HC Substance-related words - HC Neutral words))**. Voxels size = 3 × 3 × 3 mm^3^, *p* < 0.05 FWE (family wise error) corrected. **(A)** IGD subjects show increased activation in inferior parietal lobule, middle occipital gyrus and dorsolateral prefrontal cortex (DLPFC) than HC group in trials involved in gaming-related words minus neutral ones. **(B)** Correlation between RT (attentional bias: the mean RT of gaming-related words minus those of the neutral ones) and peak inferior parietal lobule activation among IGD subjects. **(C)** Correlation between RT (attentional bias: the mean RT of gaming-related words minus those of the neutral ones) and peak middle occipital gyrus activation among IGD subjects. **(D)** Correlation between RT (attentional bias: the mean RT of gaming-related words minus those of the neutral ones) and peak DLPFC activation among IGD subjects.

The blood oxygen level-dependent (BOLD) data for attention bias demonstrated significant between-group differences. Greater BOLD signals were found in the inferior parietal lobule, middle occipital gyrus and DLPFC in the gaming-related words relative to the neutral words, when comparing the IGD subjects to the HC group during the addiction Stroop process ((IGD Substance-related words - IGD Neutral words) - (HC Substance-related words - HC Neutral words)) (Figure 2A; Table 2).

**Table 2:** **Regional brain activation for the contrast of ((IGD Substance-related words - IGD Neutral words) - (HC Substance-related words - HC Neutral words))**.

**x, y, z^a^**	**Hemisphere**	**Peak intensity**	**Number of voxels^b^**	**Region^c^**	**Brodmann's area**
51, -36, 54	R	3.661	178	Inferior Parietal Lobule	40
51, -66, -15	R	3.473	135	Middle Occipital Gyrus	19
30, 30, 27	R	3.209	120	Dorsolateral Prefrontal Cortex	9

a Peak MNI coordinates.

b We first identified clusters of contiguously significant voxels at an uncorrected threshold p < 0.05, as also used for display purposes in the figures. We then tested these clusters for cluster-level FWE correction p < 0.05 and the AlphaSim estimation indicated that clusters with 90 contiguous voxels would achieve an effective FWE threshold p < 0.05. Voxel size = 3 × 3 × 3 mm^3^.

c The brain regions were referenced to the software Xjview (http://www.alivelearn.net/xjview8) and double checked with atlas.

### Correlation analyses

We analyzed the correlation between brain activity and attention bias (defined as the mean RT of the gaming-related words minus those of the neutral ones) in IGD subjects. The correlation between attentional bias and brain activity in the inferior parietal lobule among IGD subjects is 0.313 (*p*=0.192) (Figure 2B). The correlation between attentional bias and brain activity in the middle occipital gyrus among IGD subjects is 0.468 (*p*=0.043) (Figure 2C). The correlation between attentional bias and brain activity in the DLPFC among IGD subjects is 0.066 (*p*=0.789) (Figure 2D).

## Discussion

In behavioral performance, consistent with previous studies which have demonstrated that attentional bias toward substance-related words across different types of addictions, such as alcohol use, smoking and gambling (Janes et al., 2010; Marhe et al., 2013), the present study revealed that participants' responses for gaming-related words were significantly longer than that of neutral words. It suggested that heightened gaming-related salience could result in undesired distraction and cognitive interference caused by the word content, rendering them slower to respond. However, since the accuracy rates were all over 90% among all the participants in present study, it is hard to claim whether the cognitive interference caused by executive control the positive feedback of the executive control. Besides, inconsistent with previous, IGD group did not show statistical difference on RT compared with HC group. Though, we have tried our best to control the familiarity of the gaming-related words and neutral ones between the two groups, this may be an important fact could affect the un-significant result. The online gaming represents a great deal of diversity in regards to the types of games played (Chen et al., 2008). Researches have proved that the attentional bias related to gaming-related stimuli may vary across subjects depending on their preferences and frequency of playing a particular game (Peters and Malesky, 2008). And the majority of previous studies, which have demonstrated an attentional bias in related addiction, have used generalized addiction-related stimuli but failed to find any special impact of personalized stimuli in the influence of generalized alcohol-related stimuli (Fridrici et al., 2013). Thus, the un-significant attentional bias in behavioral in IGD subjects may result from the familiarity on the material among the participants. In addition, previous researchers have demonstrated that frequent gaming may contribute to improvement of performance on tasks related to visual, selective attentional abilities and motor skills (Green and Bavelier, 2003; Boot et al., 2008), in which participants have to response as fast as possible. In present study people should press the corresponding button as fast as possible. Thus, another explanation may be that the accelerated reaction can weaken attentional bias toward gaming-related words, resulting in less significant difference between RT in gaming-related words and neutral ones.

Although we observed the un-significant behavioral results, however, in neuroimaging results, this study revealed several important findings that could deepen our understanding about the neural activities in IGD. We observed greater brain activation in IGD subjects relative to the HC group in the inferior parietal lobule, the middle occipital gyrus and the DLPFC during the addiction Stroop task. Our findings are in accordance with researchers using other addicted groups such as substance addiction and pathological gambling (Dannon et al., 2011; DeVito et al., 2012). Current theories of the inferior parietal lobule function suggest that the activation is involved in sustained attention to an important stimulus feature in the face of more salient and misleading stimulus features (Hassabis et al., 2007; Lutz and Widmer, 2014). A number of studies have demonstrated that parietal regions are activated during attention shifts when attention is reflexively drawn to prominent features of a stimulus (Chelazzi and Corbetta, 2000) and have been implicated in word reading (Vossel et al., 2006). In present study the participants had to perform a simple cognitive task (color-naming task) while salient but distracting information (gaming-related words) in the environment. Therefore, present finding (IGD subjects show higher brain activation in inferior parietal lobule) might suggest that the IGD subjects experienced more cognitive conflicts and need more (top-down) attentions during the addiction Stroop task (Peterson et al., 1999; Mitchell, 2005).

The DLPFC was demonstrated to be mainly related to cognitive control processing (MacDonald et al., 2000; Milham et al., 2003), word reading/production (Carpenter et al., 2000) and working memory (Ainslie, 1975; Odum et al., 2000; Alliance, 2013; Augustus Diggs et al., 2013). It has also implicated in response inhibition/interference (Casey et al., 1997; Pujol et al., 2001). Cognitive control and working memory capacity are critical in determining performance on the Stroop task (Grant et al., 1996), which affects attention (Lecrubier et al., 1997). Therefore, the higher activation in the DLPFC in IGD is in accordance with our hypothesis that the IGD subjects have to employ more cognitive resources in controlling their automatic response toward the semantic content of the words. In other words, the IGD subjects should engage extra endeavor to control their attention from gaming-related words to accomplish the primary task (color-naming) well due to their uncontrolled desire for recognizing the semantic of the words, which resulting from long-term experience of playing games online.

A significant positive correlation was found between attentional bias and activation of middle occipital gyrus in IGD subjects, which suggested that the more endeavors were engaged toward gaming-related words, the higher the brain activities in middle occipital gyrus. The occipital activity may be associated with word reading and visual attention processes (Tang et al., 2006). And it is demonstrated that the performance of the Stroop color-word task depends on three major processes including word reading, color naming and interference resolution (Adleman et al., 2002). Therefore, the activation of middle occipital gyrus might suggest the gaming-related words have attracted IGD subjects' attention during the addiction Stroop task. More importantly, occipital and parietal brain regions are thought to be critical in visual functions (Hu et al., 2013). The occipital gyrus is involved in visual process (Tam et al., 2008; Kojima and Suzuki, 2010; Dong et al., 2012). The parietal lobe also plays a role in visual attention (Yin, 1978; Yin and Mountcastle, 1978). As for the features of IGD, the activation in visual related regions might be altered by the experience of long-time game playing.

## Conclusions

In summary, the current study shows that the gaming-related words are more interference to IGD subjects and they need to engage more endeavors to perform better for their attentions paid to the semantic rather than the color of the gaming-related words during the addiction Stroop task. More importantly, present study reveals that several abnormalities of brain regions might be implicated in underlying the pathophysiology of IGD in terms of neural results.

## Author contributions

YZ programmed the experiment, analyzed the data, and wrote the first draft of the manuscript; XL and HZ contributed to data collection, and XL, JX revised and improved the manuscript. GD designed this research, revised and improved the manuscript. XD contributed to fMRI data collection. All authors contributed to and have approved the final manuscript.

## Funding

The funders had no role in study design, data collection and analysis, decision to publish, or preparation of the manuscript.

### Conflict of interest statement

The authors declare that the research was conducted in the absence of any commercial or financial relationships that could be construed as a potential conflict of interest.
