# Supplementary material for: Brain Activity toward Gaming-Related Cues in Internet Gaming Disorder during an Addiction Stroop Task
Source: Front Psychol. 2016 May 19;7:714. doi: 10.3389/fpsyg.2016.00714 (PMC4872468; doi:10.3389/fpsyg.2016.00714)
Supplement: Supplementary file 1 [file DataSheet1.doc]

**Gaming-related words**

**Green：**

**裂魂人、黑黄杖、先锋盾、熊战士、镇魂石、末日守卫、灰烬之灵、死亡先知、振奋宝石、雷神之锤、**

**Red：**

**碎骨锤、双头龙、骷髅头、钢背兽、不朽盾、天鹰之城、支配头盔、熊猫酒仙、地狱领主、艺人面罩**

**Yellow：**

**黯灭刀、刷新球、食尸鬼、敌法师、玄铁锤、幻影刺客、王者之戒、纷争面纱、速度之靴、飞行使者**

**Neutral words**

**Green：**

**大理石、大陆架、操作台、落地窗、矮脚蕉、修辞手法、交通工具、名言警句、循环系统、经济效益**

**Red：**

**洗衣粉、出租车、普通话、文具盒、圆珠笔、承上启下、人民公园、公共汽车、中央广场、核糖核酸**

**Yellow：**

**地形图、积雨云、百叶窗、气压表、地磁场、分门别类、成语接龙、纸本水墨、磁赤铁矿、空气湿度**
